# LIPS database with LIPService: a microscopic image database of intracellular structures in *Arabidopsis* guard cells

**DOI:** 10.1186/1471-2229-13-81

**Published:** 2013-05-16

**Authors:** Takumi Higaki, Natsumaro Kutsuna, Seiichiro Hasezawa

**Affiliations:** 1 Department of Integrated Biosciences, Graduate School of Frontier Sciences, The University of Tokyo, Kashiwanoha, Kashiwa, Chiba 277-8562, Japan; 2 Advanced Measurement and Analysis, Japan Science and Technology Agency (JST), Chiyoda-ku, Tokyo 102-0076, Japan

**Keywords:** 3-D structures, *Arabidopsis thaliana*, Fluorescent proteins, Microscopic image, Organelle, Stomata

## Abstract

**Background:**

Intracellular configuration is an important feature of cell status. Recent advances in microscopic imaging techniques allow us to easily obtain a large number of microscopic images of intracellular structures. In this circumstance, automated microscopic image recognition techniques are of extreme importance to future phenomics/visible screening approaches. However, there was no benchmark microscopic image dataset for intracellular organelles in a specified plant cell type. We previously established the Live Images of Plant Stomata (LIPS) database, a publicly available collection of optical-section images of various intracellular structures of plant guard cells, as a model system of environmental signal perception and transduction. Here we report recent updates to the LIPS database and the establishment of a database table, LIPService.

**Description:**

We updated the LIPS dataset and established a new interface named LIPService to promote efficient inspection of intracellular structure configurations. Cell nuclei, microtubules, actin microfilaments, mitochondria, chloroplasts, endoplasmic reticulum, peroxisomes, endosomes, Golgi bodies, and vacuoles can be filtered using probe names or morphometric parameters such as stomatal aperture. In addition to the serial optical sectional images of the original LIPS database, new volume-rendering data for easy web browsing of three-dimensional intracellular structures have been released to allow easy inspection of their configurations or relationships with cell status/morphology. We also demonstrated the utility of the new LIPS image database for automated organelle recognition of images from another plant cell image database with image clustering analyses.

**Conclusions:**

The updated LIPS database provides a benchmark image dataset for representative intracellular structures in *Arabidopsis* guard cells. The newly released LIPService allows users to inspect the relationship between organellar three-dimensional configurations and morphometrical parameters.

## Background

Recent advances in imaging equipment and probes have enabled the acquisition of huge numbers of bioimages. One researcher can easily inspect a few hundred images. However, when the number of images increases to thousands, millions, or billions, no individual could process them alone. Cell imaging techniques in particular yield large-scale systematic image data characterizing gene and protein localizations [1]. In these situations, computer assistance becomes increasingly important for image inspection. To develop and evaluate computer programs for image inspection, a benchmark image dataset is crucial. For this purpose, the benchmark dataset should not be a miscellaneous image collection but systematically-captured images designed with a precise aim. For example, the MitoCheck consortium focused on identifying human genes involved in mitosis progression and collected two days of time-lapse images of 67 average HeLa cells expressing histone-GFP with a chemically synthesized short interfering RNA (siRNA) knockdown system with 21,000 protein-coding genes [2]. This time-lapse image dataset is freely available (http://www.mitocheck.org/). In addition, Dr. Murphy’s laboratory at Carnegie Mellon University acquired HeLa cell images with fluorescently-labeled cell nuclei, nucleoli, endoplasmic reticulum (ER), Golgi bodies, lysosomes, mitochondria, plasma membrane, endosomes, actin microfilaments, and microtubules, and proposed organelle recognition algorithms for the HeLa image dataset [3]. Benchmark images of 80–91 HeLa cells for every 10 markers are freely available at Dr. Murphy’s laboratory website (http://murphylab.web.cmu.edu/data/2Dhela_images.html). Public release of these image datasets will contribute to the future development of computational image analytics.

In the plant sciences, a few fluorescent microscopic image databases have focused on intracellular structures, such as Plant Cell Imaging (http://deepgreen.stanford.edu/) [4], the Illuminated Plant Cell (http://www.illuminatedcell.com) [5], and the Plant Organelles Database (http://podb.nibb.ac.jp/Organellome/) [6]. These databases have provided beautiful pictures of plant cell structures and cellular dynamics and have potential value as resources for model analysis [7]. However, these image databases contain images of different plant cell types, complicating analysis.

Guard cells of plant stomata have become a model system for characterizing signal transduction mechanisms from environmental perception to turgor movement [8]. Previous biological studies of guard cells have proven that intracellular structures are required for healthy stomatal movement [9-11]. To comprehensively visualize guard cell intracellular dynamics during stomatal movement, we have already released microscopic images of 50–60 pairs of *Arabidopsis* guard cells fluorescently labeled with 18 kinds of organelle markers in the Live Images of Plant Stomata (LIPS) database [12]. However, with the first version of the database, visualizing intracellular three-dimensional configurations and/or mining biologically meaningful information such as the relationship between intracellular configuration and stomatal apertures was inconvenient.

We have updated the LIPS database with additional datasets. The original serial optical sections are still available as LIPS dataset I, and volume-rendering and aligned-image datasets are newly released as LIPS datasets II and III, respectively. In addition, a database table, named LIPService, was newly established to easily inspect the relationship between intracellular configuration and cell status/morphology. In this article, we describe the updated content and utility of LIPService. This database will serve as an image data mining tool, a web-based educational resource, and a benchmark dataset for plant intracellular structures in plant guard cells.

## Construction and content

*Arabidopsis thaliana* plants were grown on soil-vermiculite in growth chambers at 23.5°C, at 60% relative humidity, and with a 12/12-h light/dark cycle using 100 μmol m^-2^ s^-1^ white lights. Epidermal strips of fully-expanded rosette leaves of 4–5-week-old plants were placed onto the inverted platform of an fluorescent microscope (IX70; Olympus, Tokyo, Japan) equipped with an oil immersion objective lens (UplanApo 100×/1.35 oil; Olympus), a spinning disc confocal unit (CSU10; Yokogawa, Tokyo, Japan), and a cooled CCD camera (CoolSNAP HQ; PhotoMetrics, Tucson, AZ, USA). GFP/YFP imaging used 488 nm excitation lasers (HPU-50101-PFS2; Furukawa, Tokyo, Japan) and 524–546 nm emission filters (FF01-535/22-25; Semrock, Rochester, NY, USA). For RFP imaging, 561 nm excitation lasers (85-YCA-025-040; CVI Melles Griot, Albuquerque, NM, USA) and 524–546 nm emission filters (FF01-590/20-25; Semrock) were used. Serial optical sections (0.5 μm intervals) were taken with Metamorph 7.0 software (Molecular Devices; Sunnyvale, CA, USA). All raw images were 500 × 500 pixels (32 × 32 μm). These fluorescent markers were chosen based on reliability and validity in previous plant cell biological studies. All transgenic plants used in the LIPS database had healthy growth and stomatal movements with a diurnal cycle [12]. We did not capture obviously abnormal guard cells with artifacts because of mechanical damage in sample preparation. To make volume-rendering data, we used an ImageJ plug-in 3D volume viewer (in ImageJ menu Plugins-3D-3D Viewer; http://3dviewer.neurofly.de/) and converted data into animated GIFs with ImageJ (http://rsbweb.nih.gov/ij/). For aligned images, we used our original KBI ImageJ plugins. For detailed procedures, please refer to our protocol [13]. The images and HTML files are located on our in-house web server. The search function is implemented with Google Custom Search (http://www.google.com/cse/). The LIPService uses Google Fusion Tables (http://support.google.com/fusiontables/).

## Utility and discussion

**Figure 1:**
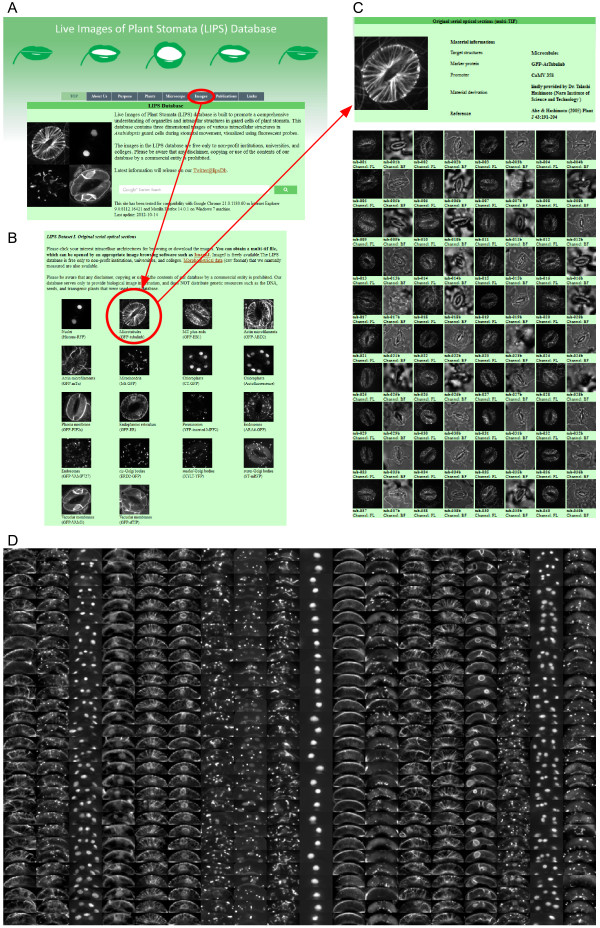
**Sample images of the LIPS database website.** (**A**) Main page of the LIPS database. Clicking on the “Images” tab (circled in red) links to thumbnails of each fluorescent probe (**B**). (**B**) Clicking on a thumbnail (circled in red) allows access to the page of the user-choice intracellular structure (e.g., microtubules) (**C**). (**C**) The microtubules page of LIPS dataset I. By clicking a thumbnail of a bright field or fluorescent image, the user can download the raw images. (**D**) Tiled aligned images in LIPS dataset III.

Detailed contents of the original LIPS image dataset were previously reported [12]. Here, we focus on how to use the LIPS database website and the new additional content. LIPS database website visitors can find image and/or literature data by entering a query word (e.g., microtubule) in the search box on the home page. To obtain a LIPS image dataset, users should visit the “Images” page (http://hasezawa.ib.k.u-tokyo.ac.jp/lips/images.html; Figure 1A). There, users will see links to the three types of image datasets, as describe below.

### Dataset I. Original serial optical sections

**Figure 2:**
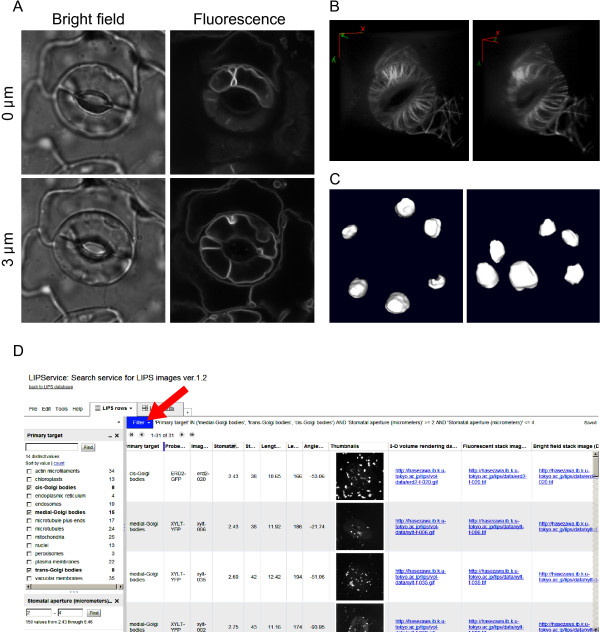
**Example applications of LIPS database images.** (**A**) Sample images from LIPS dataset I. Bright field and fluorescent images of *Arabidopsis* guard cells expressing vacuolar membrane marker GFP-VAM3 at different focal planes (0 and 3 μm). All image sizes are 500 × 500 pixels (32 × 32 μm). (**B**) Volume-rendering images of microtubules labeled with GFP-tubulin. The right and left images show different viewing angles. The 360°-rotation animations are available in LIPS dataset II. (**C**) Surface images of chloroplasts labeled with CT-GFP. The right and left images show different viewing angles. (**D**) Interface of the LIPService. Clicking on the “Filter” dropdown list (arrow) allows users to choose the “Filter” items (e.g., Primary target). After specifying the filter conditions, the user will see a list with a link to download image datasets I and II (in blue).

Dataset I is a collection of original optical sections of 18 kinds of fluorescent markers, which are listed in our previous report [12]. Users can select organelles using the tiled thumbnails (Figure 1B) and visit the user-choice page (Figure 1C). On this page, users can download the raw serial optical section 16-bit TIFF images by clicking on the thumbnails. To browse the downloaded images, we recommend using an image visualization tool such as ImageJ, which is freely available. For an overview of image visualization tools, please refer to the review by Walter et al. [1]. Representative examples of LIPS images are shown in Figure 2A. This dataset contains raw images that can be processed and analyzed with appropriate image analytic tools [1]. For example, three-dimensional models can be reconstructed from the raw serial sections with ImageJ plugins 3D Viewer (in ImageJ menu Plugins-3D-3D Viewer; Figure 2B, C).

### Dataset II. Volume rendering data

Dataset I is a raw serial optical-section dataset that can be used to reconstruct three-dimensional models with appropriate image processing software. However, such three-dimensional reconstruction requires skill and labor. To overcome this problem, Dataset II allows easy viewing of the three-dimensional organizations of the target intracellular structures. The volume rendering data (Figure 2B) from all 930 pairs of guard cells are available. The original images are 180 frames that play back at 10 frames/sec. Users can easily examine the 360°-rotation GIF animations by simply clicking on the link in the web browser, without specific image visualization tools. We believe that this feature will be a valuable aid to education.

To more efficiently search and browse the images in LIPS datasets I and II, we prepared a database interface, named LIPService (Figure 2D). Users can visit the LIPService website from the “Images” page (http://hasezawa.ib.k.u-tokyo.ac.jp/lips/images.html). On this page, users can choose the “Filter” items of interest such as “Primary target” and “Stomatal aperture” from the dropdown list (Figure 2D, arrow). When users specify the filter condition, matching thumbnail images and links to download image datasets I and II appear (Figure 2D). We believe that the LIPService will be useful for image data mining, especially in studying the relationship between intracellular configuration and morphometrical parameters, such as stomatal pore width (aperture) or length.

### Dataset III. Aligned images for localization analyses

Dataset III is a collection of aligned maximum intensity projections of the fluorescent serial optical sections. The step-by-step image processing with ImageJ used to make dataset III was described in our recent protocol paper [13]. All guard cell regions were aligned to a mean size of 304 × 119 pixels (19.5 × 7.6 μm). The fluorescent intensities were also normalized to an average of 0 with a standard deviation of 1. On the “Images” page, users can download dataset III as a ZIP file (52 MiB) containing 1,860 32-bit TIFF files (100–120 examples × 18 probes) with probe-name tags in the file names. Part of dataset III is presented as a tile in Figure 1D.

**Figure 3:**
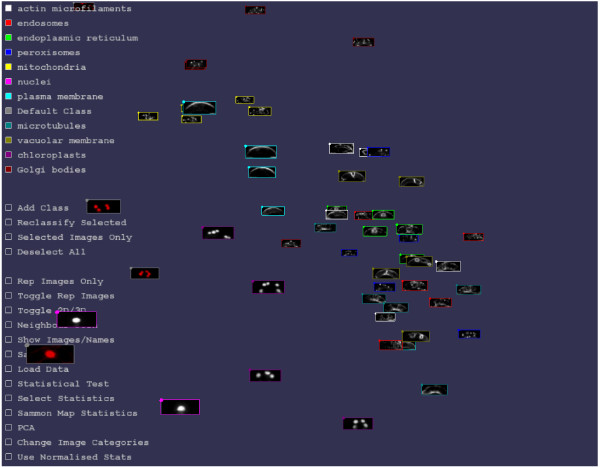
**Screenshot of iCluster as an example of image clustering results.** The six images extracted from the Plant Organelle Database [6] (Additional file 1: Figure S1) and the 55 images from LIPS dataset III were analyzed with a two-dimensional Sammon map, a clustering algorithm, implemented in iCluster software. The six Plant Organelle Database images are highlighted in red. See also Additional file 1: Figure S2.

With dataset III, users can inspect their own fluorescence images of guard cells. To demonstrate this utility, we performed image clustering analysis of images obtained from another database, the Plant Organelle Database (http://podb.nibb.ac.jp/Organellome/) [6], using guard cell images of nuclei labeled by GFP fusions with a nuclear localization sequence (NLS-GFP; Additional file 1: Figure S1A), mitochondria labeled by DsRed fusions with the pre-sequence of the delta-prime subunit of mitochondrial F1-ATPase (F1-ATPase-δ-DsRed; Additional file 1: Figure S1B), and chloroplasts labeled by GFP fusions with CAS, a thylakoid membrane-localized protein (CAS-GFP) [14] (Additional file 1: Figure S1C). After preprocessing (Additional file 1: Figure S1D-H), image clustering analysis was performed using the freely-available image clustering software iCluster (http://icluster.imb.uq.edu.au/) [15] (Figure 3, Additional file 2: Movie S1) and the 55 selected LIPS images (5 examples × 11 probes) that users can download as a ZIP file (860 KiB). iCluster gathered images of the same organelles (Figure 3), including nuclei (NLS-GFP *vs.* HistoneH2B-RFP; Additional file 1: Figure S2A), mitochondria (F1-ATPase-δ-DsRed *vs.* Mt-GFP; Additional file 1: Figure S2B), and chloroplasts (CAS-GFP *vs.* autofluorescence; Additional file 1: Figure S2C). These data show the potential usefulness of this dataset for users’ on-demand localization analyses.

## Conclusion

In this article, we describe how to use the LIPS database website and additional datasets. The newly released volume-rendering dataset II will be useful for education. In addition, the aligned image dataset III provides a useful benchmark dataset for developing an automated plant organelle recognition/classification. Using the LIPS dataset, in fact, we have already reported a framework for image data mining of intracellular dynamics during stomatal movement [12] and improving an automated classification of open/closed stomatal states [16]. We believe the updated LIPS database is a model service to allow biological researchers without specialized knowledge of database system development to easily publish their own imaging data.

## Availability and requirements

LIPS database is freely available at http://hasezawa.ib.k.u-tokyo.ac.jp/lips/index.html. The latest information is continuously released from the LIPS Twitter account (https://twitter.com/lipsDb).

## Abbreviations

ER: Endoplasmic reticulum; GFP: Green fluorescent protein; LIPS: Live Images of Plant Stomata; RFP: Red fluorescent protein.

## Competing interests

The authors declare that they have no competing interests.

## Authors’ contributions

TH planned the image database, conducted all microscopic imaging, prepared the database website, performed image clustering analyses, and wrote the manuscript. NK developed KBI ImageJ plugins and helped with database design. SH supported the project. All authors read and approved the final manuscript.

## Supplementary Material

Additional file 1: Figure S1 Preprocess for clustering analysis of fluorescent guard cell images using LIPS images. (**A-C**) Images used in this study. These images were downloaded from the Plant Organelle Database (http://podb.nibb.ac.jp/Organellome/) [6]. GFP fusions with a nuclear localization sequence (NLS-GFP) labeled nuclei (**A**); DsRed fusions with the pre-sequence of the delta-prime subunit of mitochondrial F1-ATPase (F1-ATPase-DsRed) labeled mitochondria (**B**); and GFP fusions with CAS, a thylakoid membrane-localized protein (CAS-GFP), labeled chloroplasts [14] (**C**). (**D-H**) Workflow of the preprocess for image clustering. Image. (A) was used as an example. First, the image was rotated (**D**) and cropped with a bounding box (**E**, **F**). For color images, only the target channel was selected to obtain a gray-scale image. The cropped images were then interpolated to the same size as in dataset III, and their intensities normalized to an average intensity of 0 with a standard deviation of 1 (**G**). After similar processing, the one-sided guard cells were rotated 180° (upside down) (**H**). **Figure S2.** Magnified images of the iCluster clustering results. Note that the same organelle images, including nuclei (**A**), mitochondria (**B**) and chloroplasts (**C**), were gathered in the three-dimensional Sammon map. Please also see Additional file 2: Movie S1. Click here for file

Additional file 2 **Movie S1.** Three-dimensional Sammon map of the iCluster clustering results shown in Figure 3. Click here for file
