# Supplementary figures and images for: LIPS database with LIPService: a microscopic image database of intracellular structures in Arabidopsis guard cells
Source: BMC Plant Biol. 2013 May 16;13:81. doi: 10.1186/1471-2229-13-81 (PMC3663689; doi:10.1186/1471-2229-13-81)

Higaki et al.  
Supplementary Figure 1.

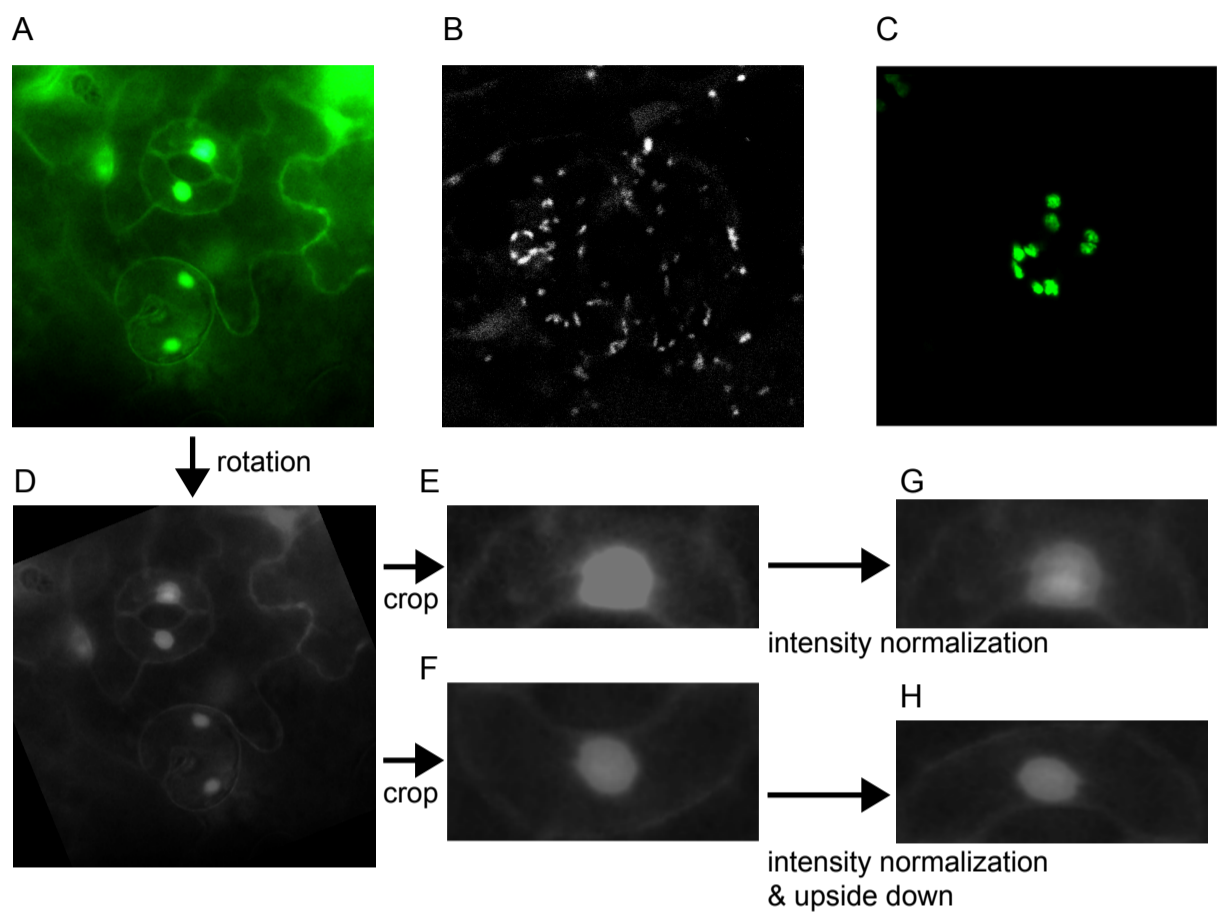

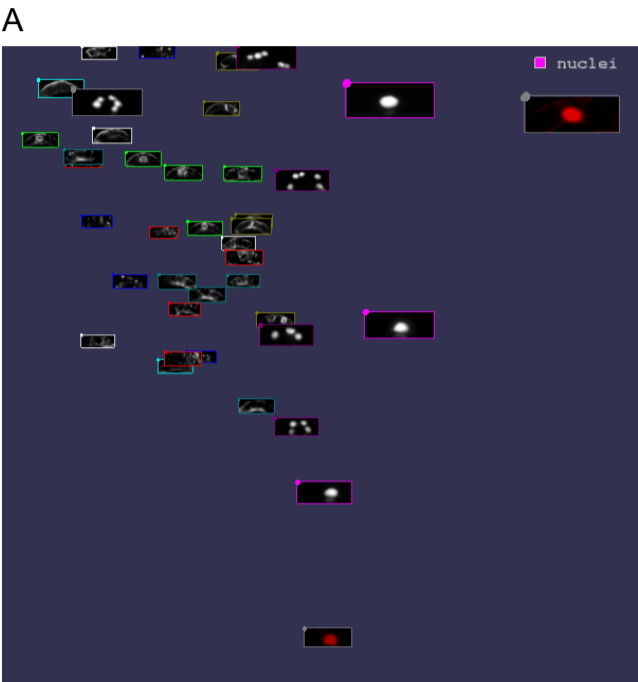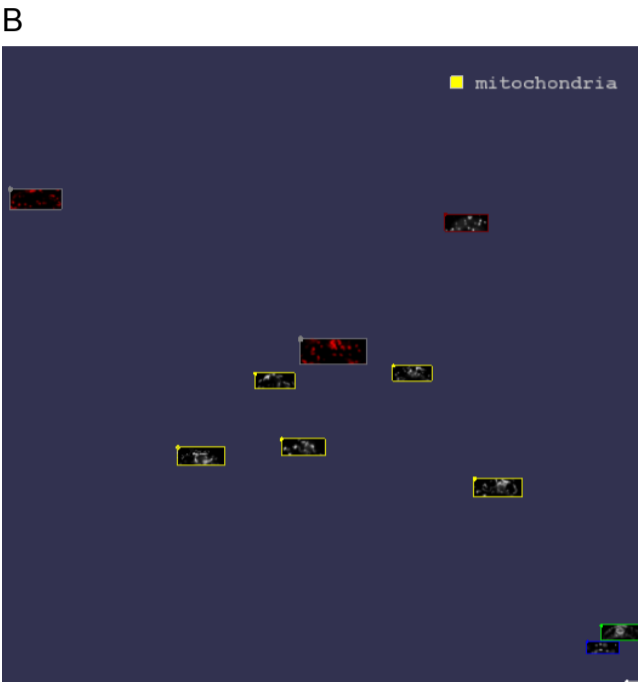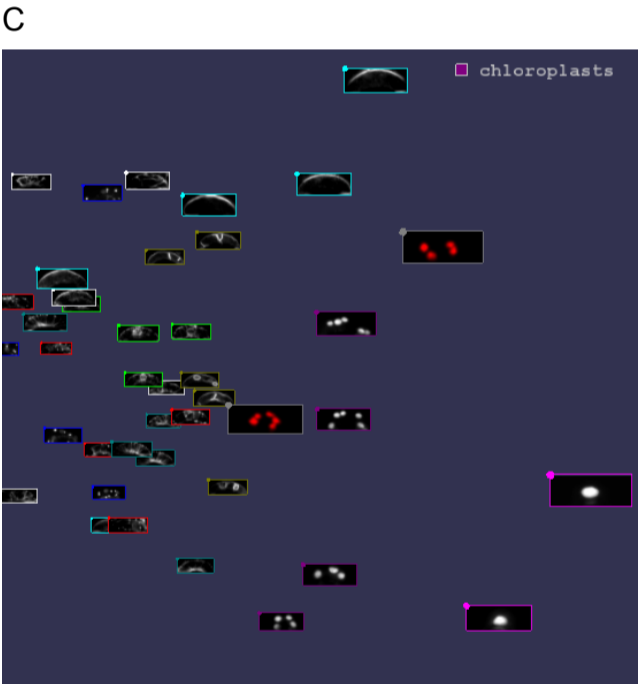

Supplement: Additional file 1: Figure S1 — Preprocess for clustering analysis of fluorescent guard cell images using LIPS images. (A-C) Images used in this study. These images were downloaded from the Plant Organelle Database (http://podb.nibb.ac.jp/Organellome/) [6]. GFP fusions with a nuclear localization sequence (NLS-GFP) labeled nuclei (A); DsRed fusions with the pre-sequence of the delta-prime subunit of mitochondrial F1-ATPase (F1-ATPase-DsRed) labeled mitochondria (B); and GFP fusions with CAS, a thylakoid membrane-localized protein (CAS-GFP), labeled chloroplasts [14] (C). (D-H) Workflow of the preprocess for image clustering. Image. (A) was used as an example. First, the image was rotated (D) and cropped with a bounding box (E, F). For color images, only the target channel was selected to obtain a gray-scale image. The cropped images were then interpolated to the same size as in dataset III, and their intensities normalized to an average intensity of 0 with a standard deviation of 1 (G). After similar processing, the one-sided guard cells were rotated 180° (upside down) (H). Figure S2. Magnified images of the iCluster clustering results. Note that the same organelle images, including nuclei (A), mitochondria (B) and chloroplasts (C), were gathered in the three-dimensional Sammon map. Please also see Additional file 2: Movie S1. [file 1471-2229-13-81-S1.pdf]
